# Novel Activators of the Transcription Factor PPARγ for Beef Quality through Molecular Modeling

**DOI:** 10.1021/acsomega.5c08474

**Published:** 2025-11-20

**Authors:** Artur G. Nogueira, Daiana T. Mancini, Taináh M. R. Santos, Teodorico C. Ramalho

**Affiliations:** Department of Chemistry, Federal University of Lavras, Lavras, MG 37200-000, Brazil

## Abstract

Tropical beef has low marbling and variable intramuscular lipids, making the meat appear between dry and juicy due to the lack of intramuscular fat. The objective of this work is to obtain the 3D structure of bovine PPARγ and discover new compounds that activate it. The target sequence used showed high sequence identity with the human PPARγ protein model. Virtual screening and molecular docking identified compounds that activate human PPARγ. The docking parameters were validated through ROC curve and redocking, and a virtual screening resulted in 274 compounds for further research. After applying filters, 12 compounds showed better affinity with PPARγ and lower toxicity compared with the reference ligand. Compound 3 and Compound 4 showed favorable interactions with PPARγ in molecular dynamics simulations, raising expectations for the discovery of new activators.

## Graphical abstract

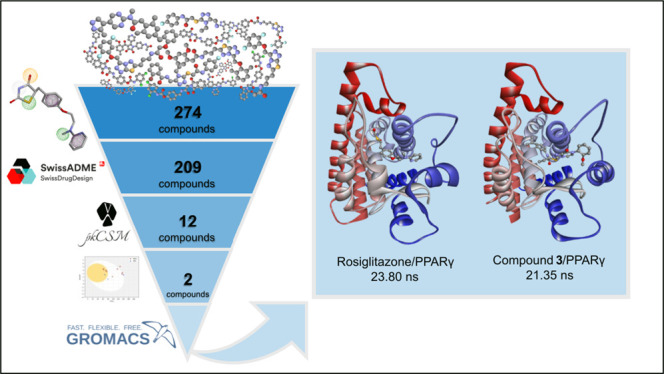

## 1 Introduction

The meat economy plays a significant role in several areas, including the economy, food, culture, and environmental issues. The bovine cattle have been presented as one of the most valuable meats along with meats of pigs,^1^ as shown in Figure 1, attesting to their preference by consumer taste. According to de Viacava, Carlos, tropical meat is practically devoid of marbling, where the results of hundreds of analyses reveal a range of 1.5–3.5% of intramuscular lipids in sirloin steaks. The juiciness of the meat varies from dry to succulent, due to the lack of intramuscular fat, which corroborates that tropical meat is treated as a regular commodity.^2–4^

**Figure 1:**
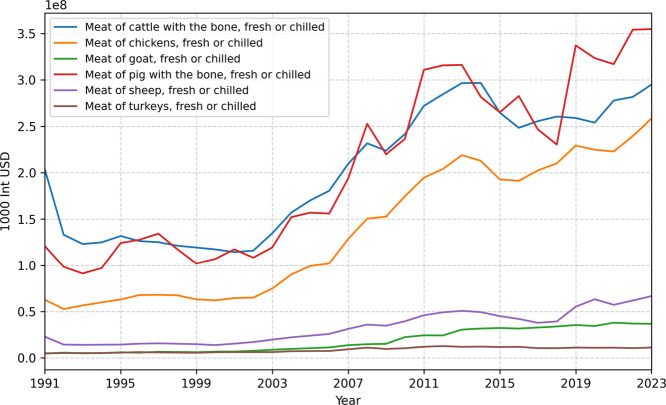
Value of meat production between 1991 and 2023. The data represented can be found in the Value of Agricultural Production (Gross Production Value (current thousand USD)) section on the FAOSTAT platform.

In Brazil, most of the herd is made up of zebu cattle, mainly of the Nelore breed (*Bos taurus indicus*). The meat from these animals is generally less tender and has less marbling compared to meat from taurine cattle, especially the Angus breed (*Bos taurus taurus*).^2,3^ This characteristic reduces the attractiveness of zebu meat since tenderness and marbling are considered the main attributes of palatability by consumers.^5,6^ Marbling refers to the amount of intramuscular fat and is considered an important characteristic because it is directly related to the sensory characteristics of the meat, which can be appreciated by the consumer.^7^

The animal body’s lipid reserve is represented by a state of equilibrium between fat synthesis and catabolism. Once the balance is destroyed, fat deposition is increased or decreased^8^ to re-establish the previous balance. According to Hausman, subcutaneous, internal, seam, and intramuscular adipose tissue deposits are all economically and physiologically important in meat animal production, where it is claimed that deciphering the regulation of each adipose deposit can lead to new animal production strategies.^9^

In this context, adipogenesis occurs in two main stages: (A) determination, which involves the commitment of a pluripotent stem cell to the adipogenic lineage. During this stage, the stem cell is converted into a preadipocyte that is morphologically indistinguishable from its precursor but has lost the potential to differentiate into other cell types and (B) terminal differentiation, in which the preadipocyte acquires the typical features of a mature adipocyte, including the machinery required for lipid transport and synthesis, insulin sensitivity, and the secretion of adipocyte-specific proteins.^10^

Several transcription factors are essential for promoting adipogenesis, among which peroxisome proliferator-activated receptor γ (PPARγ) and CCAAT/enhancer-binding proteins (C/EBPs) play central roles. A key difference between them is that PPARγ alone can stimulate the differentiation of preadipocytes into mature adipocytes, clearly demonstrating its critical role in adipogenesis.^11^ Furthermore, PPARγ activation promotes terminal differentiation by inducing a series of genes involved in triglyceride uptake and storage, such as fatty acid-binding protein (aP2), acyl-CoA synthetase, fatty acid transport protein-1, lipoprotein lipase, and others.^10,12,13^

Although PPARγ is the central regulator of adipogenesis, the process is complex and involves several additional transcription factors, such as C/EBPα, KLFs, and STATs. These factors typically act upstream to induce PPARγ expression or cooperate with it to activate the adipogenic gene program. However, PPARγ remains indispensable, as neither these factors alone nor in combination can drive adipogenesis in the absence of PPARγ activity.^14,15^

In addition, como a proteína PPARγ have the power to regulate the expression of various genes affecting lipid and carbohydrate metabolism.^16^ One of the main strategies used to synthesize adipocytes in vitro is supplementation with an adipogenic differentiation cocktail^17^ to increase beef marbling. This protocol consists of a combination of three or four adipogenic inducers that are usually added to the medium in three phases: induction, progression, and maintenance.^18^ Insulin is commonly used in combination with the synthetic differentiation inducers dexamethasone, 3-isobutyl-1-methylxanthine (IBMX), and rosiglitazone.

Rada Mitić and colleagues investigated the complete exclusion and three different concentrations of each component (rosiglitazone, insulin, IBMX, and dexamethasone). Removing insulin or rosiglitazone from the traditional protocol resulted in significantly decreased differentiation compared to the control, revealing its necessity.^18^ Rosiglitazone is an antidiabetic drug belonging to the thiazolidinedione class, acting as an insulin sensitizer through its binding to peroxisome proliferator-activated receptors (PPARγ) in adipose cells, thereby enhancing cellular responsiveness to insulin. However, a meta-analysis conducted by Steven E. Nissen and Kathy Wolski, published in The New England Journal of Medicine in 2007, associated the use of rosiglitazone with an increased risk of adverse cardiac events, including myocardial infarction.^19^ In addition, rosiglitazone is toxic and therefore not compatible with food.^19^

Thus, the objective of our work is to propose an adipogenic compound, given that rosiglitazone will be replaced by a PPARγ (peroxisome proliferator-activated receptor γ) agonist that is less toxic and therefore suitable for safely inducing fat production in culture.

## 2 Methods

### 2.1 Transcription Factor Target Selection and Modeling by Homology

Homology modeling was used to predict the three-dimensional structure of bovine PPARγ. For this purpose, a known and experimentally validated human PPARγ (PDB ID: 1FM6 ^20^) was used as the template protein. The amino acid sequence of the bovine PPARγ used for modeling is present in the UniProt database^21^ with the code UniProtKB/SwissProt: A0A6P5DU68 of the organism *Bos taurus indicus.*

The SWISS-MODEL server^22,23^ was used to perform homology modeling. The criteria for evaluation of the constructed model were (1) sequence identity; (2) Ramachandran chart;^24^ and (3) QMEANDiscCo.^25^

### 2.2 Structure-Based Virtual Screening

The virtual screening of potential PPARγ activators was performed in the MolPort, Mcule, and ZINC molecule banks, using the Pharmit Search Engine platform (https://pharmit.csb.pitt.edu) for the first two banks and the ZINCPharmer platform for the last one. The chemical structure of rosiglitazone (RGZ) used during the virtual screening is the crystallized form together with human PPARγ (PDB ID: 1FM6).^20^ Next, filters based on the parameters described by Lipinski and co-workers were applied in order to reduce the number of compounds returned. These criteria, known as the rule of five, include up to 5 hydrogen bond donors (HBD), up to 10 hydrogen bond acceptors (HBA), molecular weight up to 500 g/mol, and logP up to 5. These filters aim to select compounds with a higher probability of absorption.^26^ The set of hits obtained was initially submitted to the SwissADME,^27^ where a qualitative toxicological analysis was performed that consisted of separating only the hits that do not have violations of the rules and parameters of Lipinski,^28^ PAINS,^29^ and Brenk^30^ simultaneously.

### 2.3 Molecular Docking Study and Analysis

To validate the use of the proposed program for molecular *docking* of the proposed ligands to PPARγ, AutoDock Vina,^31,32^ the statistical means chosen were the area under the receiver operating characteristic (ROC) curve and the molecular redocking. For the construction of the ROC curve, five active compounds (Table 2) with proven biological activity on the PPARγ were selected.^33^ From these compounds, false actives (so-called decoys) were generated in order to assess the software’s ability to distinguish between active compounds and false actives. For each of the active compounds, 50 decoys were generated using the DUD.E platform.^34^ The assessment of molecular redocking involved the calculation of the root-mean-square deviation. This value was obtained by aligning the docked structure with the crystallographic structure (rosiglitazone) through superposition.

After these validations, the sets of compounds found in the virtual screening and accepted in terms of toxicity (without alerts and violations of Lipinski, PAINS, and Brenk rules) were docked into the active site using AutoDock Vina with a total of 30 exchanges executed for each ligand. From the analysis of ligand–protein interactions, the conformation with the highest affinity was chosen. Also, the set of screening compounds was assessed for its quantitative toxicity by means of the oral rat acute toxicity (LD_50_) via the pkCSM platform.^35^

The groups of the compounds have been ranked by the best affinity values simultaneously with the lowest LD_50_ values. The top compounds ranked were evaluated for BBB permeance and P-gp substrate potential, using the BOILED-Egg chart.

### 2.4 Molecular Dynamic (MD) Simulation

Molecular dynamics (MD) simulations elucidate the temporal interatomic interactions within a protein and other molecular systems closely resembling their natural conditions. These simulations effectively capture a spectrum of biological processes, including ligand binding, complex stability, and protein folding.^36,37^ In this current investigation, molecular dynamics simulations were carried out using the software GROMACS-5.1.2.^38^ Molecular dynamics simulations were carried out on *Bos taurus indicus* PPARγ complexes with rosiglitazone and two of the highest ranked compounds, MCULE-2772386084 and MCULE-1323064686. ATB Version 3.0 (https://atb.uq.edu.au/) was used to create topology files for the ligands. The simulation was carried out in an SPC water environment, along with counterions (Na^+^/Cl^–^) to neutralize the system.

The NVT/NPT ensemble was established at a temperature of 300 K and an atmospheric pressure of 1 bar. The simulation utilized the GROMOS54a7 force field. The simulation duration was defined as 150 ns. Prior to energy minimization, the system was neutralized by adding Na^+^ and Cl^–^ ions. Energy minimization was then performed using the Verlet cutoff scheme, with Coulomb interactions treated by the particle mesh Ewald (PME) method^39^ and van der Waals interactions handled using a cutoff of 1.2 nm. The NVT equilibration was conducted for 1 ns using the V-rescale thermostat,^40^ followed by an NPT equilibration for 5 ns employing the V-rescale thermostat and the Berendsen barostat.^41^ The production phase of 150 ns was carried out under the V-rescale thermostat and the Parrinello–Rahman barostat.^42,43^ After carrying out the 150 ns molecular dynamics steps, the hydrogen bond, interaction energy, RMSD (root-mean-square deviation), and RMSF (root-mean-square fluctuation) graphs were generated and analyzed for each system.

## 3 Results and Discussion

### 3.1 Homology Modeling of PPARγ

It is important to note that the absence of a crystallographic structure of bovine PPARγ is a significant limitation in understanding the structure and function of this important molecular target. Therefore, modeling the molecular target by homology becomes a valuable tool to fill this gap. Based on the crystallographic structure of another known PPARγ, homology modeling allows the creation of a three-dimensional representation of the target protein.

The homology modeling using the human PPARγ (PDB ID: 1FM6 ^20^) and the primary sequence UniProtKB: A0A6P5DU68 of the organism *Bos taurus indicus* presented a coverage range equal to 54% (residues 234 to 505). The 272 residues present in the created model showed a sequence identity of 98.90%, with only 3 residues (SER-296, PRO-301, and ASN-330) differing from the template, as shown in Figure 2. Furthermore, Figure 2 presents the alignment between the sequences of the two structures. An important detail is that the three differing residues are not located within the protein cavity for molecular docking.

**Figure 2:**
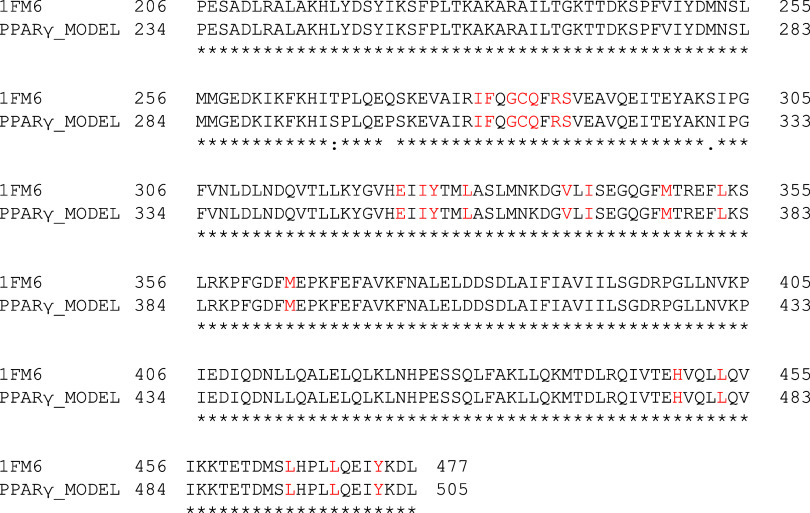
Alignment template with the modeled transcription factor. Fully conserved residues are indicated with an asterisk (*), while (:) and (.) indicate residues with strongly similar and residues with weakly similar properties, respectively. Residues marked in red are present in the cavity and inside the docking grid box.

The platform SWISS-MODEL returned a Ramachandran favored value of 92.22%. In the calculation of a Ramachandran plot (Figure 3), the atoms are treated as hard spheres whose dimensions correspond to their van der Waals radii. If a combination of the angles Φ and Ψ results in a collision of the spheres, it is considered sterically unfavorable and thus the residues are identified as outlier residues. The residues known as Ramachandran outliers in the model created are PRO-387; GLN-299; LEU-298; ASP-271; MET-491; SER-492; SER-296; ARG-385; PRO-297; and PRO-301.

**Figure 3:**
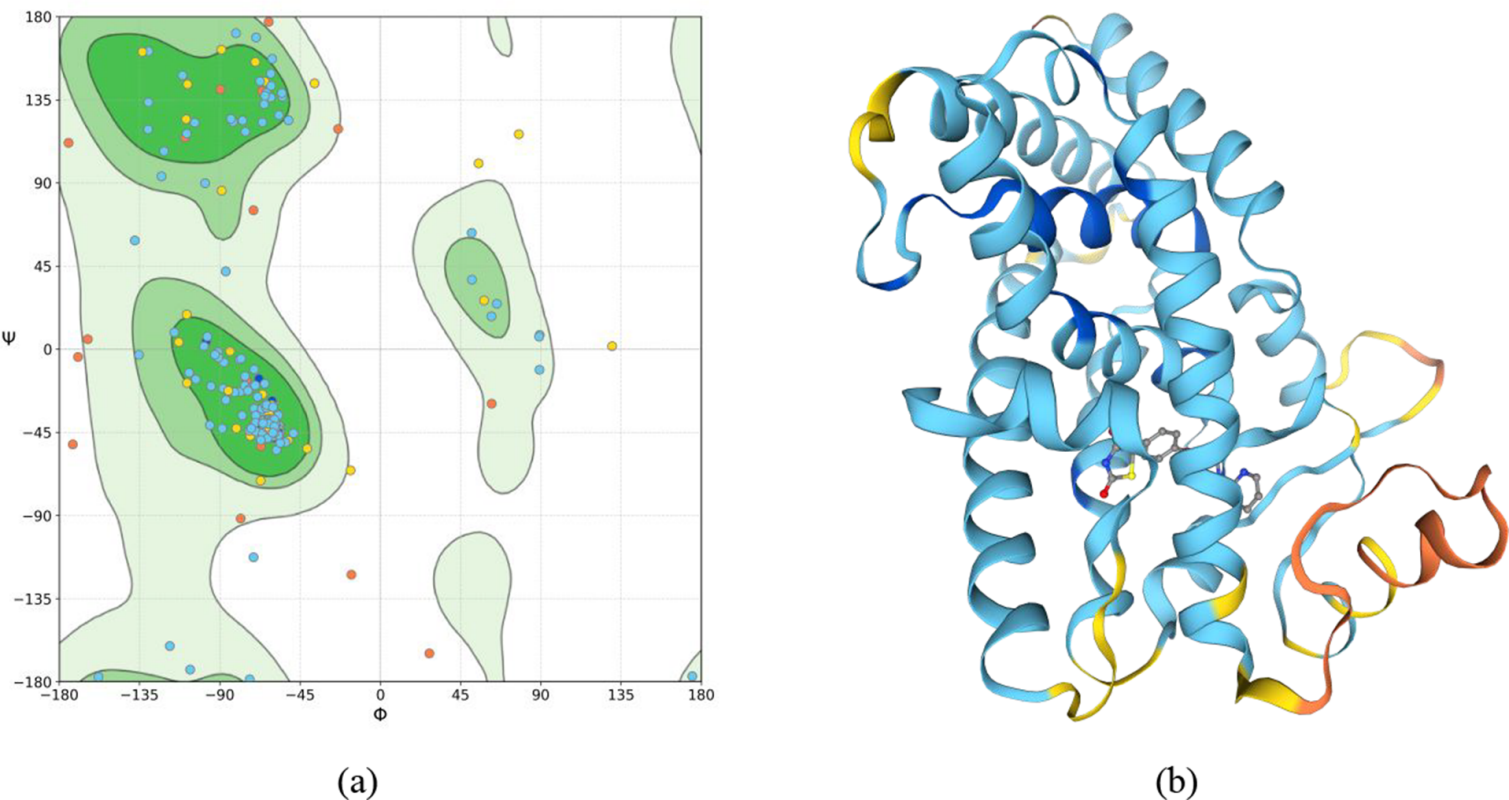
Ramachandran plot (a) and structure of the model (b). The residuals are colored by the degree of confidence based on the QMEANDisCo metric, where navy blue represents very high confidence (QMEANDisCo > 0.9), blue indicates confidence (0.7 < QMEANDisCo < 0.9), yellow indicates low confidence (0.5 < QMEANDisCo < 0.7), and orange indicates very very low confidence (QMEANDisCo < 0.5).

Among the outlier residues, five belong to the cavity that contains the rosiglitazone molecule in the human PPARγ used as the template for the modeling, which are SER-296, PRO-297, LEU-298, GLN-299, and MET-491. However, none of the outlier residues perform intermolecular interactions with rosiglitazone, as shown in Figure 4b.

**Figure 4:**
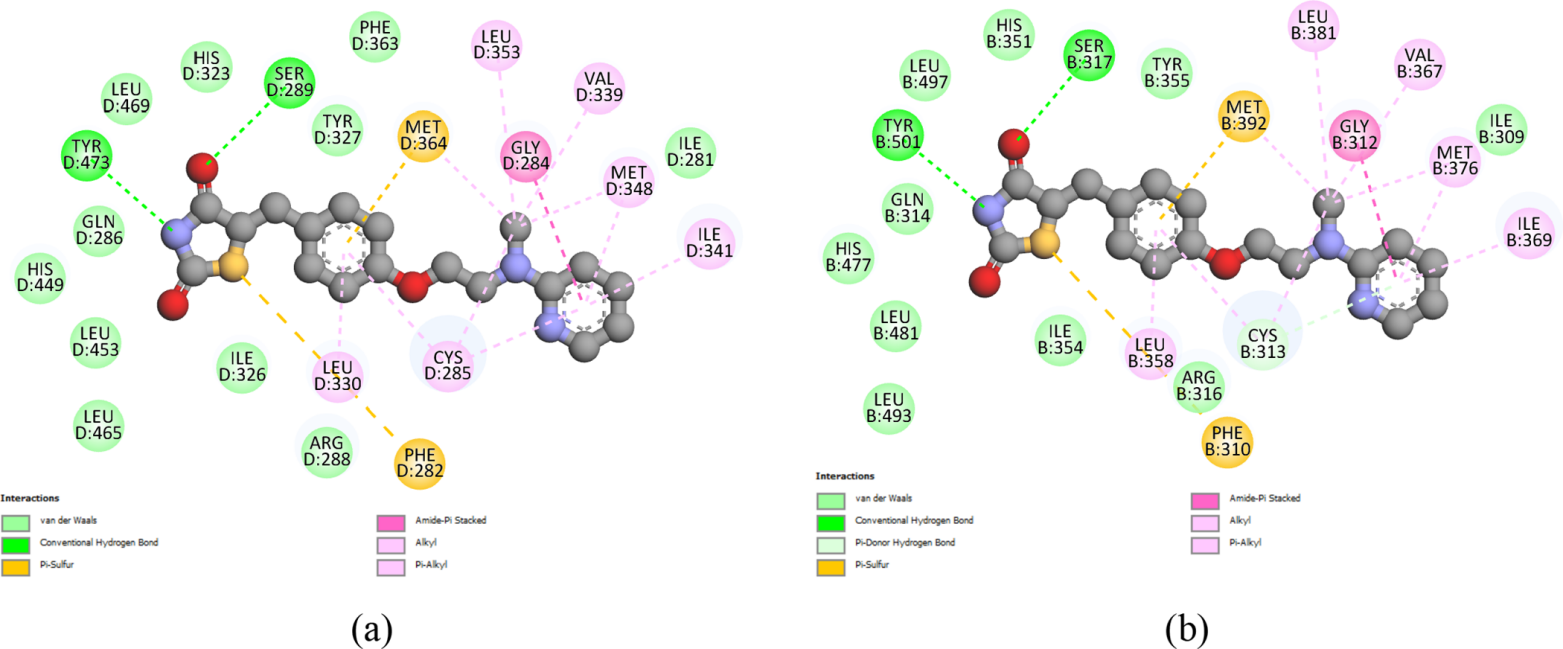
PPARγ rosiglitazone interactions on a 2D diagram: (a) 1FM6–rosiglitazone interactions; (b) PPARγ_model–rosiglitazone interactions. Both diagrams show nearly identical interactions involving the same residues; however, the residue numbering differs in (b) due to the modeling procedure used for the construction of the PPARγ_model.

The cavity to be used in the *docking* was calculated using the Cavity tool^44^ present in the platform Cavity Plus.^45^ The chosen cavity was based on the location of rosiglitazone (RGZ) crystallized in human PPAR (PDB ID: 1FM6). GMQE and QMEANDisCo provide an overall measurement of the model quality between 0 and 1, with higher numbers indicating higher expected quality. The calculation of the GMQE parameter is coverage-dependent. Thus, a model that covers only half of the primary sequence used is unlikely to score above 0.5. As expected, the GMQE found was equal to 0.44, since the coverage range was 54% (residues 234 to 505). In contrast, QMEANDisCo evaluates the current model without explicitly depending on the coverage range. After this consideration, the expectation that the QMEANDisCo value would be higher than the GMQE was fulfilled, with the QMEANDisCo value equal to 0.77 ± 0.05.

### 3.2 Virtual Screening

Virtual ligand-based screening (TVBL) is a strategy that employs compounds with known biological activity as a starting point regardless of the structure of a molecular target. This approach aims to identify molecules that have certain structural similarity and thus similar activity when interacting with the molecular target.^46^ One strand belonging to the set of TVBL approaches is searching by determining the pharmacophoric pattern.

The construction of the pharmacophore pattern used was done considering the atoms and regions of rosiglitazone that interact with PPARγ (Figure 4) and the pharmacophores considered by Justin et al. and Nanjan et al.^47^ ^,^ ^48^ The pharmacophore pattern was designed considering the thiazolidinedione ring as the acid polar head of rosiglitazone, and only the pharmacophores were selected that performed interactions with PPARγ (Figure 5), specifically (1) oxygen of the amide as the hydrogen acceptor; (2) nitrogen of the amide as the hydrogen donor; and (3) the sulfur atom as the hydrophobic group, for not having options in the platforms that best described it. Justin et al., presented the disubstituted benzene ring of the structure as an aromatic spacer, complementing the positioning. Nanjan et al., presented the same ring as a hydrophobic connector, thus the aromatic (4) and hydrophobic (5) pharmacophores were used to describe this region of the rosiglitazone molecule.

**Figure 5:**
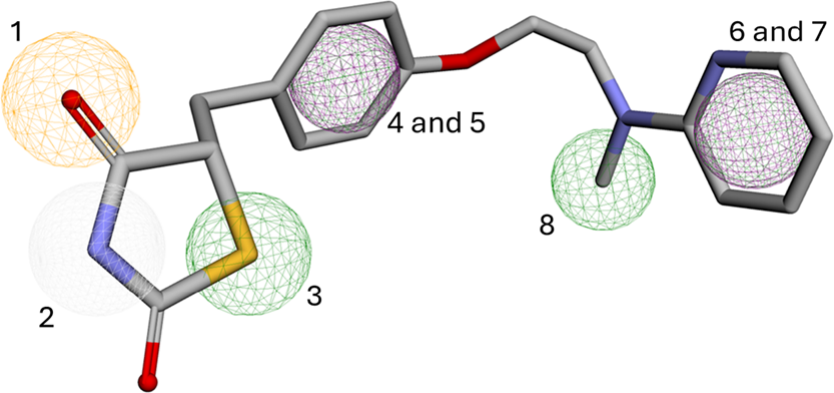
Pharmacophoric pattern of rosiglitazone. The spheres change color according to the pharmacophoric feature: orange for the hydrogen bond acceptor (1), white for the hydrogen bond donor (2), green for hydrophobic (3, 5, 6, 8), and purple for aromatic (4, 7).

To describe the other hydrophobic end effectively, the hydrophobic pharmacophore (6) was chosen, having its sphere located on the pyridine ring. On the same ring is also located the aromatic pharmacophore (7), due to the vast number of Pi interactions identified in this region. As the methyl radical located as one of the substituents of the tertiary amine performs several alky interactions, this region was described by the hydrophobic pharmacophore (8).

The libraries of molecules chosen (Table 1) under the action of the pharmacophore pattern (Figure 5) and the Lipinski rule returned 274 hits, of which 24 are repeated molecules, thus, leaving 250 hits. Among the 250 hits, only 199 were submitted to the molecular *docking* and LD_50_ calculations, as they did not present alerts (PAINS and Brenk) or violations (Lipinski), following the SwissADME system.

**Table 1:** Virtual Screening Results

web server	libraries	hits
Pharmit Search Engine	MolPort	103
	MCULE	164
ZINCPharmer	ZINC Purchasable	7

### 3.3 Validation Data

Prior to conducting the *docking* calculations using the 199 selected compounds, the AutoDock Vina program was validated. The structures ciglitazone, englitazone, pioglitazone, rosiglitazone, and didehydro rosiglitazone (5Z)- were chosen. In the context of molecular docking software validation, the AUC metric was used to summarize the overall model performance across all possible cutoff points.^49^ The AUC ranges between 0 and 1, where 0.5 represents a randomly performing model, and 1 indicates a perfect model. In our simulation, an AUC value of 0.8408 was obtained, as shown in Figure 6a. This score suggests that the molecular *docking* parameters used in AutoDock Vina software have a good ability to distinguish between the classes of true positives (Table 2) and false positives (250 decoys), indicating promising performance.

**Table 2:** Interaction Energy of Active Compounds of the Thiazolidinedione Class with the PPARγ Receptor of *Bos taurus indicus*

active compound	affinity (kcal mol^–1^)
ciglitazone	–8.0
englitazone	–9.1
pioglitazone	–8.7
rosiglitazone	–8.5
didehydro rosiglitazone (5Z)-	–8.7

**Figure 6:**
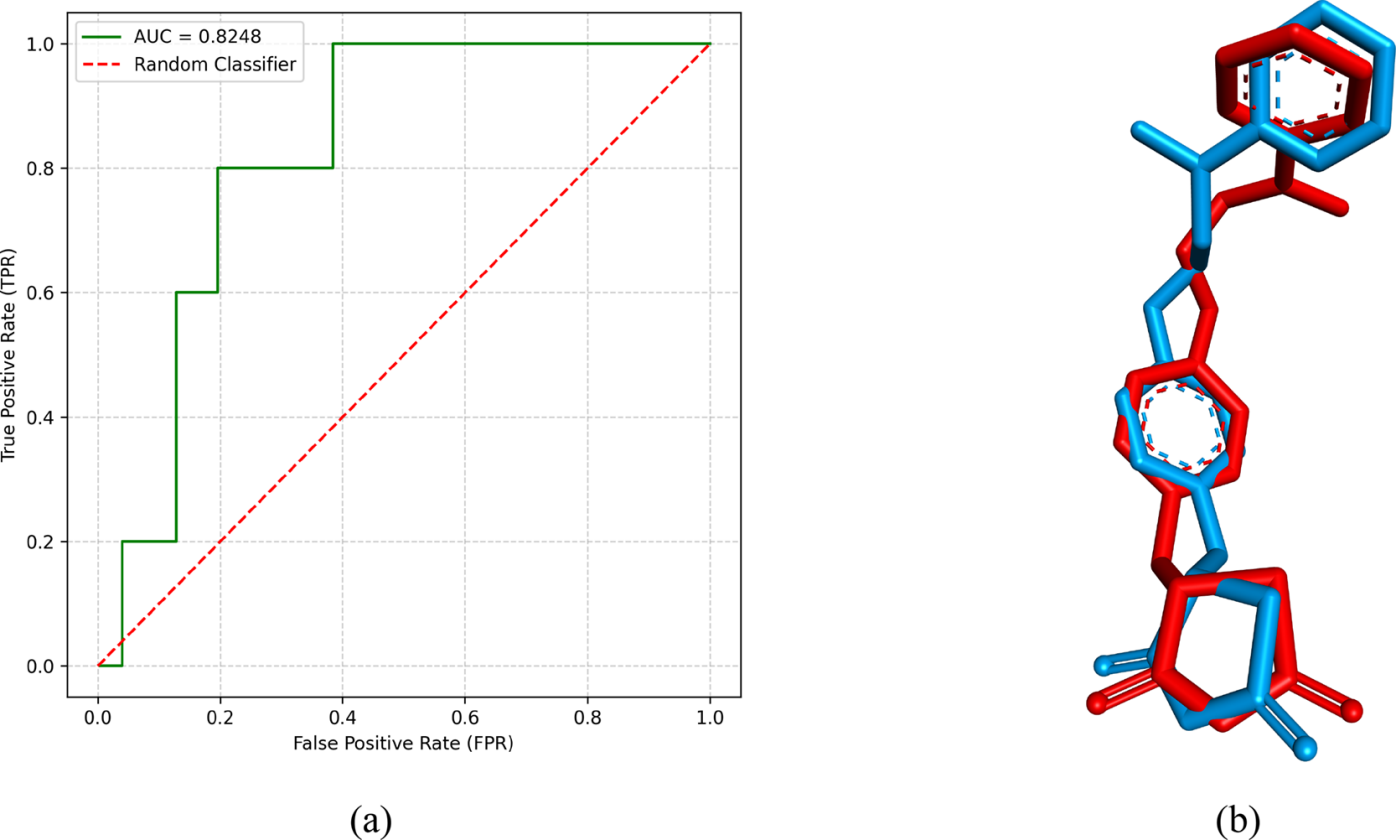
Receiver characteristics (ROC) (a) and redocking (b). In (b), Rosiglitazone crystallized is in red and rosiglitazone pose *docking* is in blue.

In this study, the DockRMSD^50^ structural superposition algorithm has been used to align the structures and calculate the RMSD. The RMSD value obtained for the two structures was 1.703 Å, indicating considerable structural similarity (Figure 6b). This value suggests that the two structures are highly overlapping with minor variations in atom positions. This low discrepancy between the structures is indicative of a possible functional and structural conservation of both rosiglitazone molecules.

### 3.4 Molecular Docking, and LD_50_ Predicted and BOILED-Egg Model

After virtual screening, the 199 compounds that did not show alerts (PAINS and Brenk) and violations (Lipinski) according to the SwissADME system were evaluated. This evaluation was carried out using the scatter plot (Figure 7), where the affinity and LD_50_ values were plotted and analyzed. Hits that are in the quadrant formed by the limits established by the affinity (≤−8.5 kcal mol^–1^) and LD_50_ (≥2.713 mol kg^–1^) values of rosiglitazone (RGZ) are considered promising, indicating prosperous affinity with bovine PPARγ and safety based on the calculated toxicity value. The molecules belonging to the quadrant were submitted to the first ranking by Euclidean distance (eq 1), where it was established that the molecules with the greatest potential are in the region with a high LD_50_ value and a large affinity modulus.

**Figure 7:**
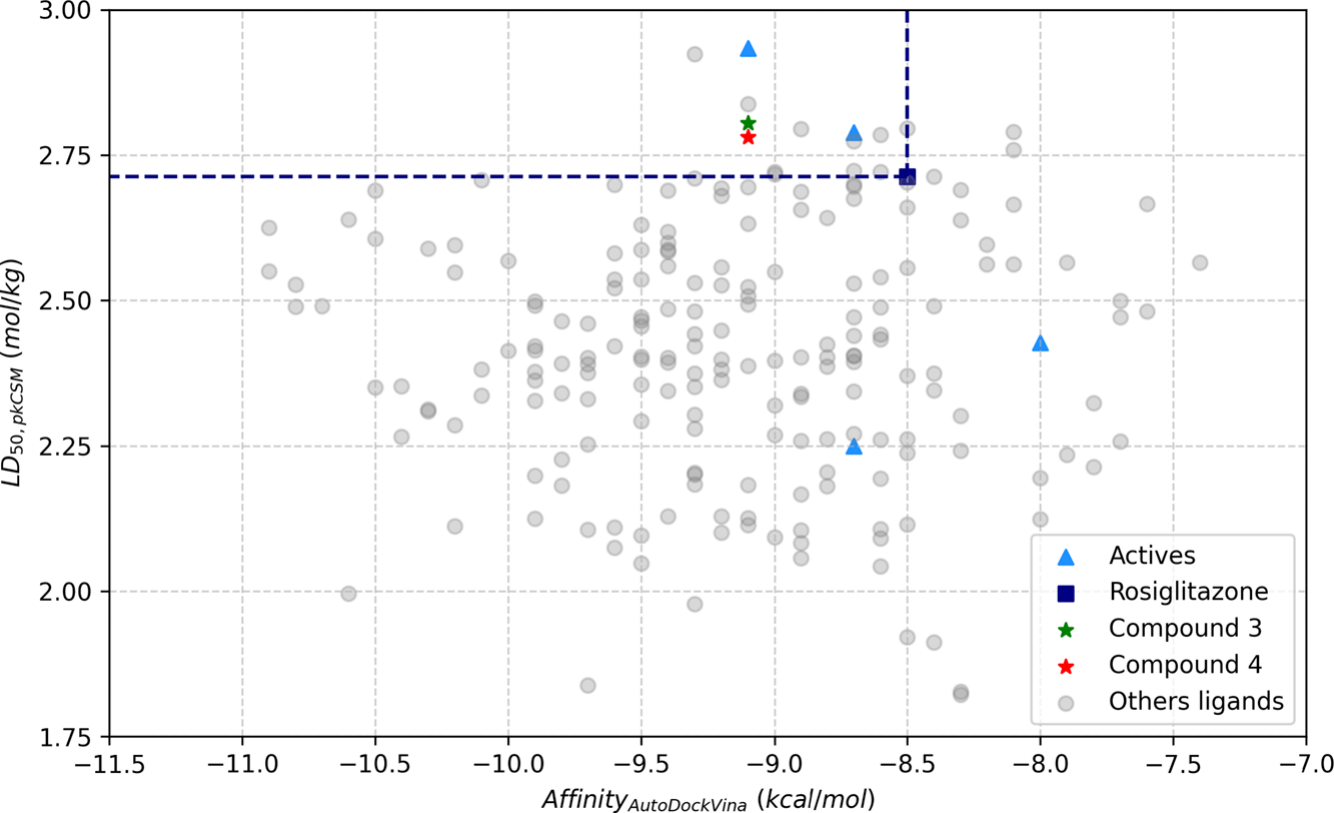
Scatter plot of affinity versus LD_5__0_ values. The dashed lines delimit the region corresponding to compounds with higher LD_50_ values (lower toxicity) and a better affinity for the PPARγ transcription factor.

The Affinity_min_ and LD_50,max_ values correspond to the most favorable affinity and toxicity results, respectively, among the 199 compounds that passed the qualitative toxicology test. The Affinity_min_ and LD_50, max_ coordinates represent the values of a hypothetical ideal compound within this set, where eq 1 is used to calculate the distance to this hypothetical point with the best affinity and toxicity values.

$${\mathrm{dist}}_{i}=\sqrt{({\mathrm{Affinity}}_{i}-{\mathrm{Affinity}}_{\min}{)}^{2}+({\mathrm{LD}}_{50}-{\mathrm{LD}}_{50,\max}{)}^{2}}$$ 1

The value of Affinity_min_ used was −11.1 kcal/mol in reference to compounds MCULE-3391486348 and MCULE-5173208549, while the value of LD_50,max_ used was 2.924 mol/kg, a value belonging to compound MCULE-9385738471. The 12 molecules located within the quadrant are sorted in the ascending order (Table 3) by the distance values calculated in eq 1.

**Table 3:** Relationship between Affinity, Toxicity (LD_5__0_), and Absorption/Permeability Properties Predicted by the BOILED-Egg Model for Rosiglitazone (RGZ) and the 12 Most Promising Compounds, Ranked According to the Euclidean Distance Presented in Eq 1

ranking	MCULE	affinity (kcal/mol)	LD_50_ (mol/kg)	BOILED-Egg region	P-gp substrate
RGZ	8293284864	–8.5	2.713	HIA	–
1	9385738471	–9.3	2.924	Out	–
2	2321610882	–9.1	2.838	BBB	+
3	2772386084	–9.1	2.805	HIA	–
4	1323064686	–9.1	2.781	HIA	+
5	1362639167	–9.0	2.721	BBB	+
6	5835167846	–9.0	2.717	Out	+
7	1302121223	–8.9	2.795	BBB	–
8	1468861772	–8.7	2.774	BBB	–
9	7373425071	–8.7	2.723	Out	–
10	9059019223	–8.6	2.785	BBB	–
11	7585853459	–8.6	2.721	HIA	–
12	8688852980	–8.5	2.796	BBB	–

The analysis of the ranking exposed in Table 3 was performed with the aid of the BOILED-Egg graph (Figure 8), where all hits belonging to the quadrant limited by rosiglitazone (Figure 7) were submitted to the calculation of pharmacokinetic property prediction (WLogP and TPSA) for plotting the BOILED-Egg graph (Figure 8). P-glycoprotein (P-gp) is encoded by the highly conserved multidrug (MDR)-resistant genes. and its overexpression can lead to multidrug resistance, rendering antibiotics and antivirals ineffective.^51–54^ Identifying substrates of P-gp prior to drug development is a promising approach to overcoming this resistance. Aiming at an absorption like that of rosiglitazone, but a better transport out of the central nervous system through P-glycoprotein, Compound **3** proved to be safer to administer than Compound **4.**

**Figure 8:**
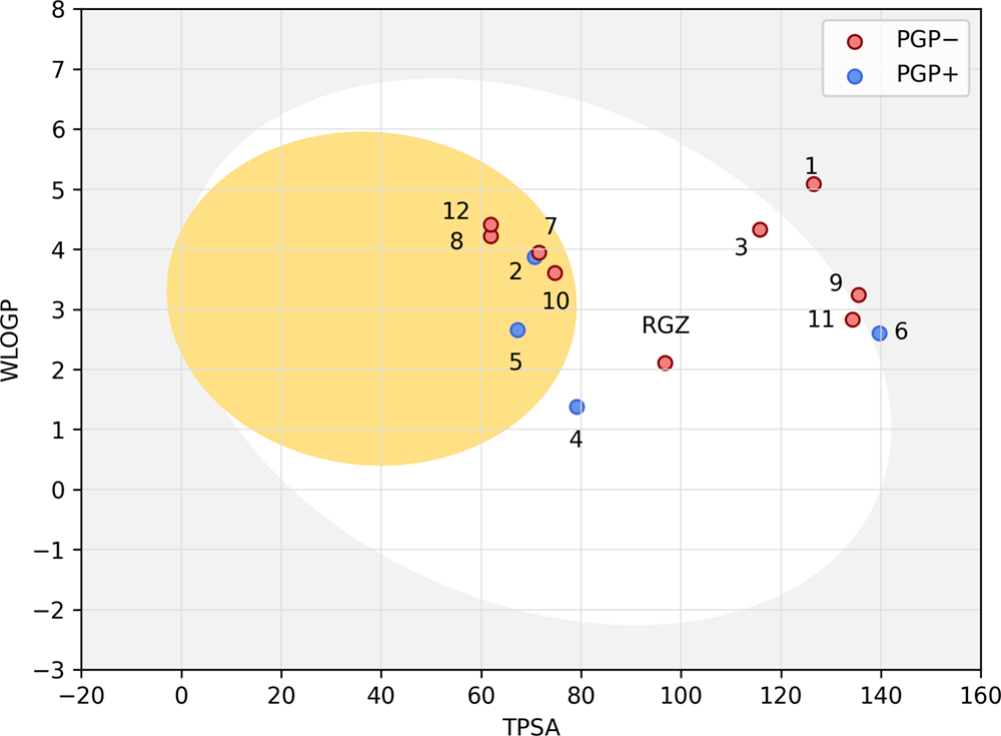
BOILED-Egg of top twelve compounds targeting PPARγ *Bos taurus indicus*. The yellow region indicates predicted blood-brain barrier (BBB) penetration, while the white region corresponds to compounds likely to be passively absorbed in the gastrointestinal tract (HIA). Compounds in the gray area are predicted to have low gastrointestinal absorption and no brain penetration. P-gp+ compounds (blue dots) are expected to be effluxed from the central nervous system by P-glycoprotein, whereas P-gp– compounds (red dots) are not.

Moreover, the overlap of Compound 3 with the pharmacophore model revealed direct correspondences with several structural elements. The oxygen and nitrogen atoms of the secondary amide acted as a hydrogen bond acceptor and donor, respectively, accurately reproducing pharmacophoric features (1) and (2). The sulfur atom of the molecule was identified as a hydrophobic group corresponding to pharmacophore (3). In addition, the 1,2,4-triazole ring contributed to both the aromatic (4) and hydrophobic (5) features. The monocyclic benzene moiety of the methoxybenzene group overlapped with pharmacophores (6) and (7), representing hydrophobic and aromatic regions, respectively. Finally, the methyl group of the ether corresponded to pharmacophore (8), reinforcing the apolar character of this terminal molecular region. The pharmacophore indices mentioned correspond to those presented in Figure 5.

Similarly, Compound 4 exhibited structural overlaps consistent with the pharmacophore model. The carbonyl oxygen and amide nitrogen located on the same side of the aryl substituent of xanthine corresponded to pharmacophores (1) and (2), acting as hydrogen bond acceptors and donors, respectively. The imidazole ring served as the hydrophobic group associated with pharmacophore (3). The aromatic and hydrophobic features (4) and (5) were assigned to the aryl group attached to the nitrogenous portion of xanthine. Furthermore, the monocyclic benzene moiety of the o-tolyl group overlapped with pharmacophores (6) and (7), while the methyl group of the same substituent represented pharmacophore (8), characterizing a terminal hydrophobic domain. As in the previous case, the pharmacophore indices refer to those shown in Figure 5.

As part of the analysis of the interactions between Compound **3** and the PPARγ receptor (Figure 9a), the 2D diagram visualizes the molecular interactions between the two entities in detail. Hydrogen bonds (H-bonds) with residues CYS-313, Pi–anion with residues GLU-287, and Pi–sulfur with residues MET-392 along with the various van der Waals interactions along the structure of Compound **3** stand out. H-bonds with residues ARG-316 and GLU-319 and Pi–sigma with residues ILE-369 stand out among the interactions between Compound **4** and PPARγ receptors.

**Figure 9:**
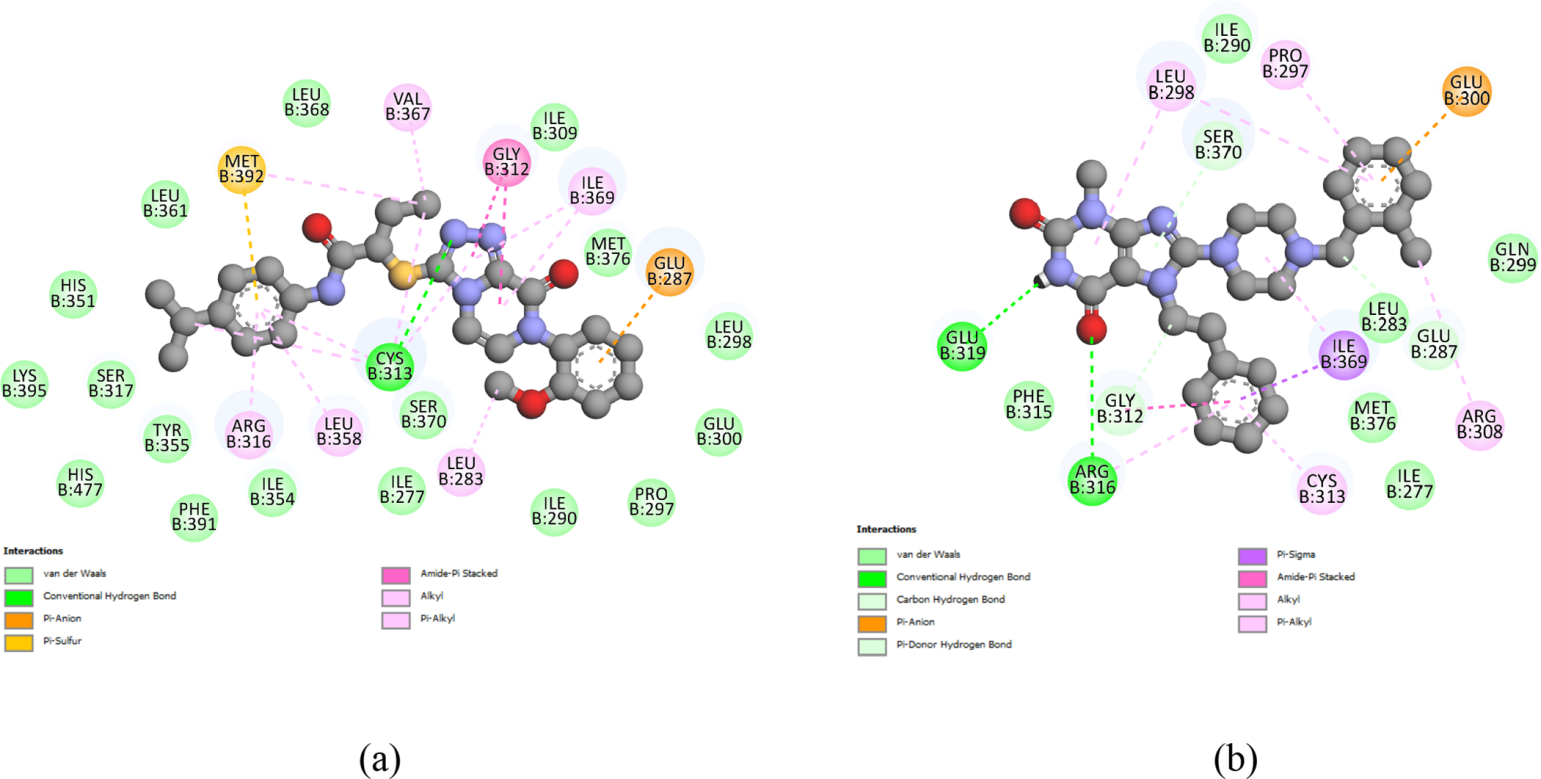
2D diagram interactions between PPARγ_model and Compound 3 (a) and PPARγ_model and Compound 4 (b). The diagram was made using the best pose of compounds in molecular *docking.*

To analyze the stability of the interactions of Compound **3** (Figure 9a), Compound **4** (Figure 9b), and rosiglitazone (Figure 4b) with the PPARγ receptor over time, a study of molecular dynamics simulation has been performed. In this line, our findings revealed a detailed analysis of the fluctuations and behavior of the residues over an extended period. Understanding how these interactions can influence the activity of the receptor and its biological effects is crucial for the development of effective pharmaceutical compounds that modulate PPARγ activity.

### 3.5 Molecular Dynamics (MD) Simulations

#### 3.5.1 Root-Mean-Square Deviation (RMSD)

RMSD calculations were carried out between the structure of PPARγ and its backbone when complexed with the structure of rosiglitazone, Compound **3,** and Compound **4**. The main objective was to assess whether there was any substantial change in the initial structure during the simulation. Figure 10 shows the RMSD results for PPARγ complexed with the respective activators. Based on the analysis of the RMSD graphs, it was found that PPARγ, when coupled with Compound **4**, showed greater stability than when complexed with Rosiglitazone or Compound **3**. When complexed with Compound **3**, the structure of PPARγ underwent greater conformational changes, because of the smaller number of strong interactions such as H-bonds, as was shown in the molecular docking results (Figure 9a). Thus, the number of H-bonds between the transcription factor PPARγ and the ligands directly interfered with its stability over the molecular dynamics simulation time. In Figure 10, the structure of PPARγ underwent constant conformational changes in the first few nanoseconds of the simulation when it complexed with any of the three ligands studied. These initial changes enabled rapid and simultaneous interactions with the ligands. After this initial phase of the major structural reconfiguration, the RMSD values remained practically stable throughout the simulation. Among the complexes analyzed, PPARγ bound to Compound **3** showed the highest RMSD value, followed by the complex with rosiglitazone, and, last, the complex with Compound **4**. This shows that PPARγ is more stable when complexed with Compound **4**.

**Figure 10:**
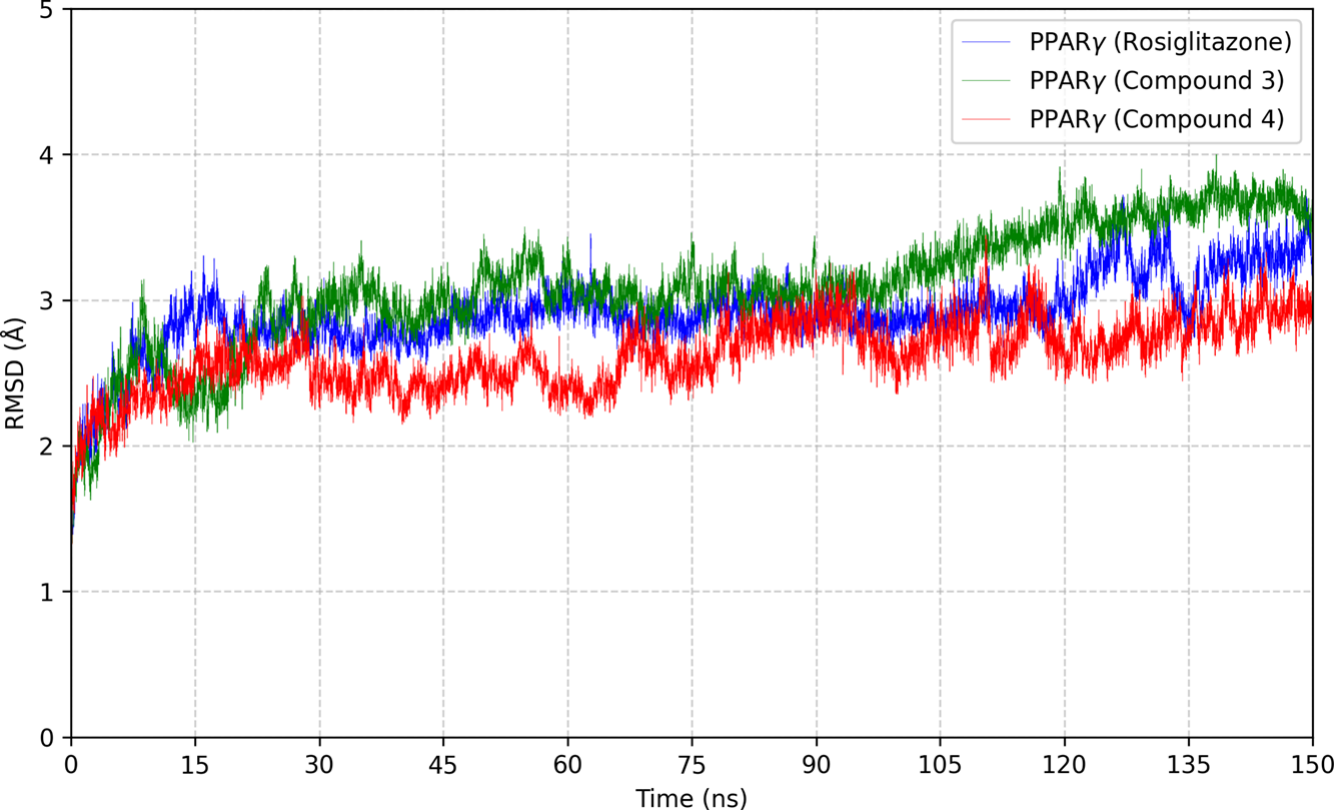
RMSD values of PPARγ were monitored over 150 ns in MD simulations.

#### 3.5.2 Root-Mean-Square Fluctuation (RMSF)

To assess structural stabilization in the simulation environment, the root-mean-square fluctuation (RMSF) was calculated based on the average position of each amino acid residue in PPARγ. Figure 11 shows the RMSF graphs for the rosiglitazone/PPARγ, Compound **3**/PPARγ, and Compound **4**/PPARγ systems.

**Figure 11:**
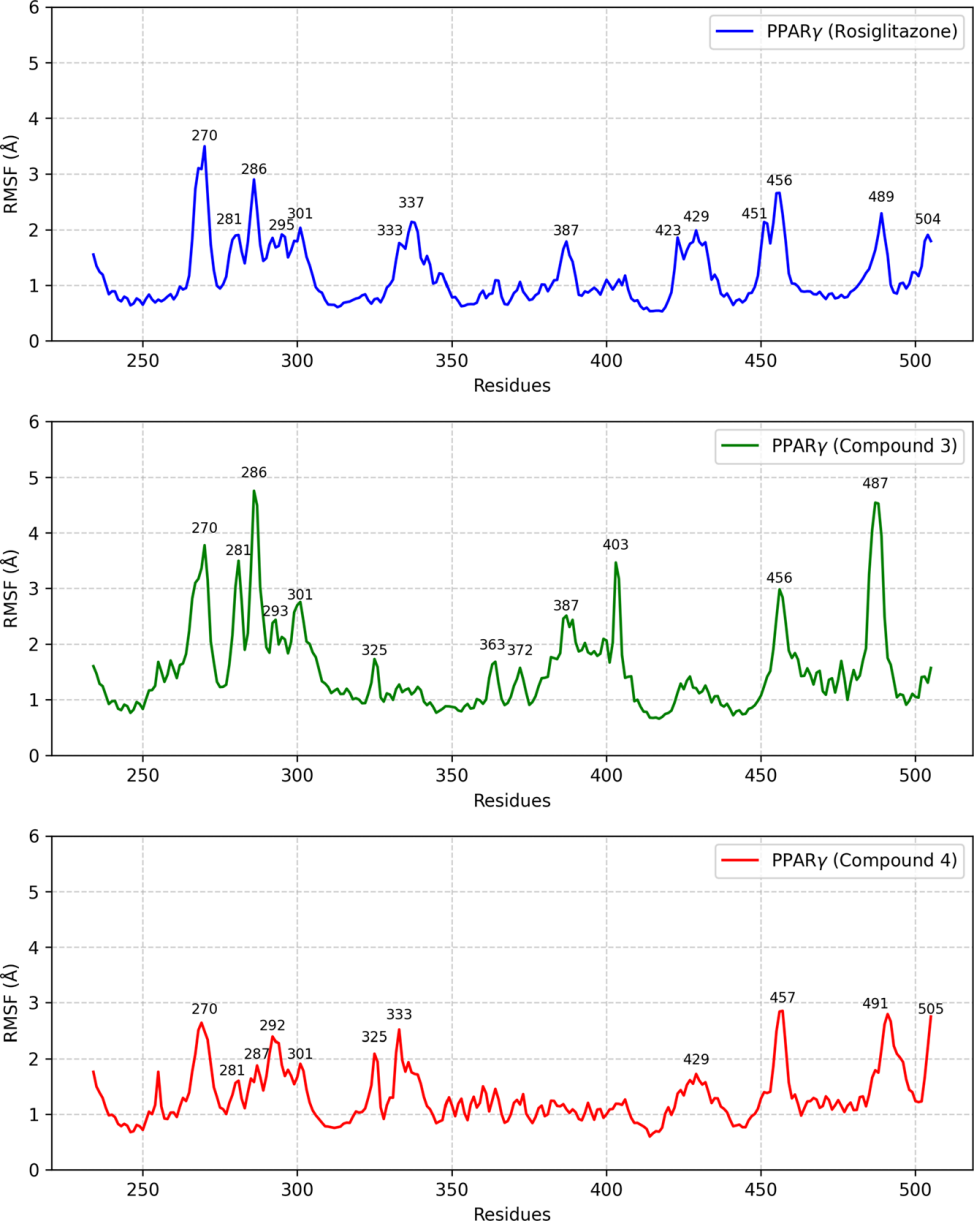
Average residual fluctuations over 150 ns in MD simulations. The analysis was specifically performed for the backbone.

From Figure 11, the higher RMSF values indicate residues that showed greater oscillation in relation to their initial positions. In the rosiglitazone system, these residues were THR-270, ASN-281, GLY-286, ILE-295, PRO-301, GLY-333, LEU-337, PRO-387, GLY-423, LEU-429, LEU-451, SER-456, THR-489, and LEU-504. In the Compound **3** system, the residues were THR-270, ASN-281, GLY-286, LYS-293, PRO-301, THR-325, ASN-363, GLY-372, PRO-387, ASN-403, SER-456, and THR-487. In the Compound **4** system, the residues were THR-270, ASN-281, GLU-287, PHE-292, PRO-301, THR-325, GLY-333, LEU-429, SER-457, MET-491, and THR-505.

Among the residues with the greatest fluctuations, the only ones present in the interaction diagrams from the molecular docking calculation were residue GLY-286 for the Compound **3**/PPPARγ complex and residue GLU-287 for the Compound **4**/PPPARγ complex. Both residues have a favorable nonclassical H-bond interaction called carbon H-bond with their respective ligands. Most of the residues with the greatest fluctuation are a part of protein loops, which are regions characterized by flexibility. Furthermore, although both systems were made up of the same protein and with the same simulation criteria, fluctuations were observed in different amino acids, possibly due to interactions with different activators.

#### 3.5.3 Analysis of Interactions in the PPARγ Active Site

In our analysis, the hydrogen bonds formed between PPARγ and the chosen ligands have been studied. A maximum donor–acceptor distance of 3.5 Å in GROMACS has been adjusted. The number of hydrogen bonds formed during the simulation is plotted in Figure 12.

**Figure 12:**
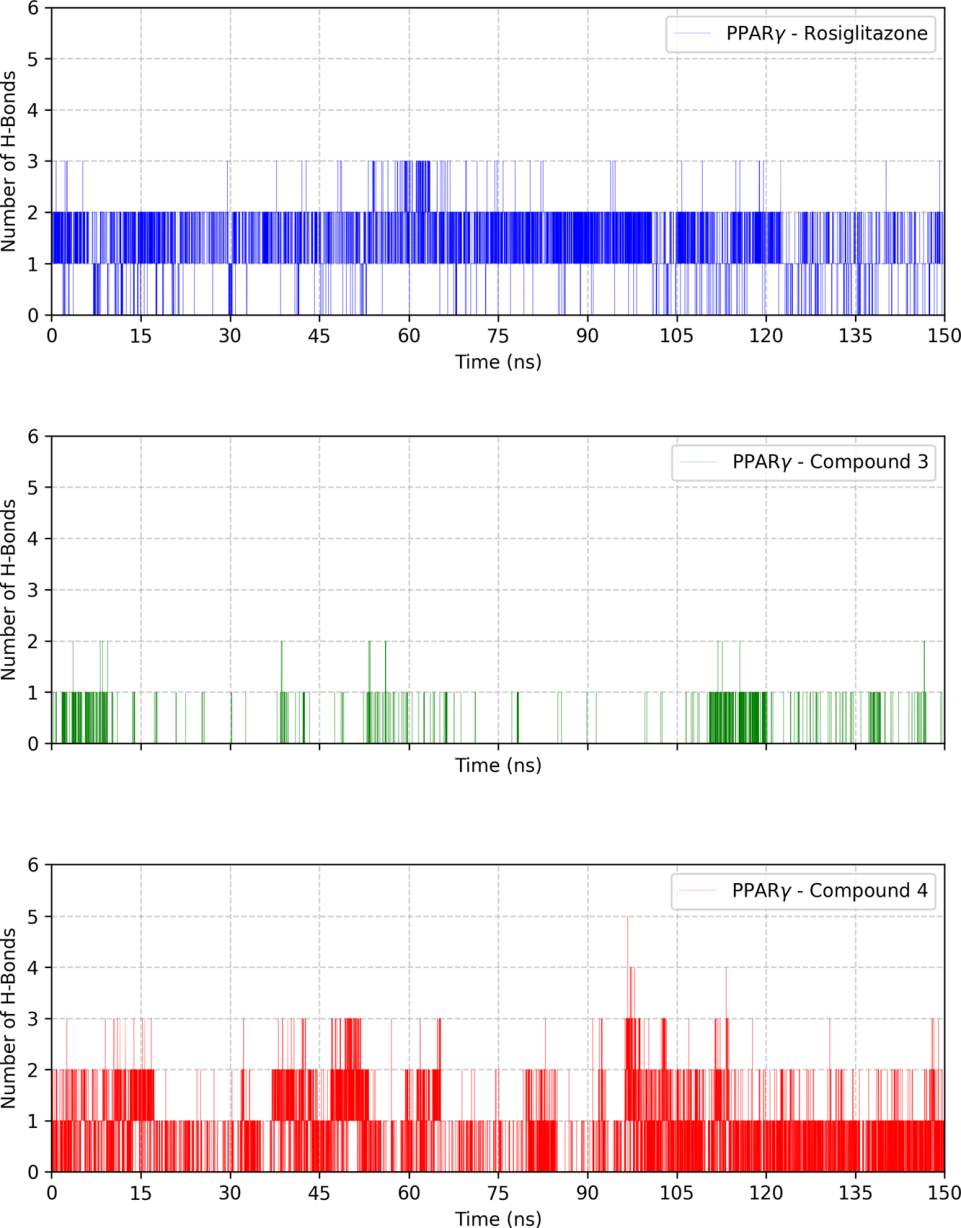
Number of hydrogen bonds between the ligands and PPARγ during the simulation period.

From the graphs, the reference ligand rosiglitazone had the most consistent hydrogen bond interactions throughout the simulation period, varying between one and five interactions. Compound **3** showed few H-bonds during the simulation period, as expected from the molecular *docking* results (Figure 9a), where at this point it is possible to see the greater instability of the transcription factor (Figure 10) over the simulation time and the greater fluctuation of the residues (Figure 11). Compound **4**, on the other hand, reached three hydrogen bonds during the simulation at sporadic moments, but more recurrently than the reference ligand, corroborating the *docking* results shown in Figures 4b and 9a,b.

To better evaluate protein–ligand interactions, GROMACS MD simulation trajectories were used to extract two crucial energy terms: short-range Coulomb energies (Coul-SR) and long-range Lennard–Jones energies (LJ-SR). Considering the short-range Lennard–Jones and short-range Coulomb potential energy calculations between the protein and the ligands, it was observed that Compound **3** and Compound **4** interact in a similar way to rosiglitazone within the PPARγ active site.

During the 150 ns simulation period, the average values of the sum of the Coul-SR and LJ-SR energies were −858,589.2 kJ/mol, −859,009.6 kJ/mol, and −859,268.4 kJ/mol for the rosiglitazone, Compound **3**, and Compound **4** ligands, respectively. These findings suggest that Compound **3** and Compound **4** can form a thermodynamically stable complex with bovine PPARγ. The ratio between the interaction energies was calculated to compare the binding potential of the compounds to the bovine PPARγ receptor. The ratio between the interaction energy of Compound 3 and rosiglitazone was 1.0005, while the ratio for Compound **4** was 1.0008. These values suggest that although both compounds have slightly more negative interaction energies than rosiglitazone, Compound **4** showed a stronger interaction with the PPARγ receptor than Compound 3. However, in relative terms, the interaction energy for both Compound **3** and Compound **4** is more satisfactory than that with rosiglitazone, indicating that these compounds may form more stable complexes with the receptor and possibly have greater potential as PPARγ modulators.

During the simulation analysis, the distribution of average interactions was examined using pharmacophore maps (Figures 13 and 14), plotted at the points of minimum interaction energy between the ligands and PPARγ. Remarkably, the H-bond interactions involving residues SER-317 and TYR-501 predicted in the molecular docking calculation were maintained throughout the molecular dynamics, as were the interactions with residues GLY-312, LEU-358, and MET-392 and other residues present in both interaction diagrams of the rosiglitazone/PPARγ system (Figure 13).

**Figure 13:**
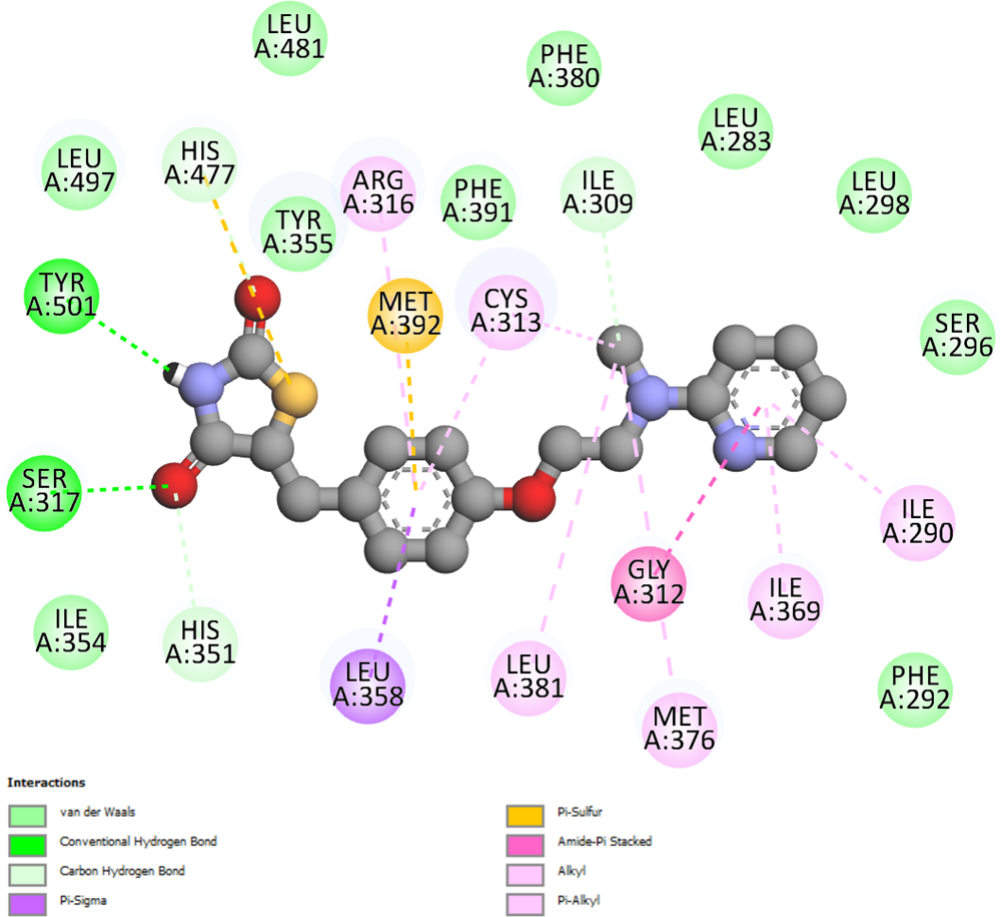
Interactions performed during 150 ns in the molecular dynamics (MD) simulation for rosiglitazone/PPARγ. The diagrams were performed on a frame of 23.81 ns.

**Figure 14:**
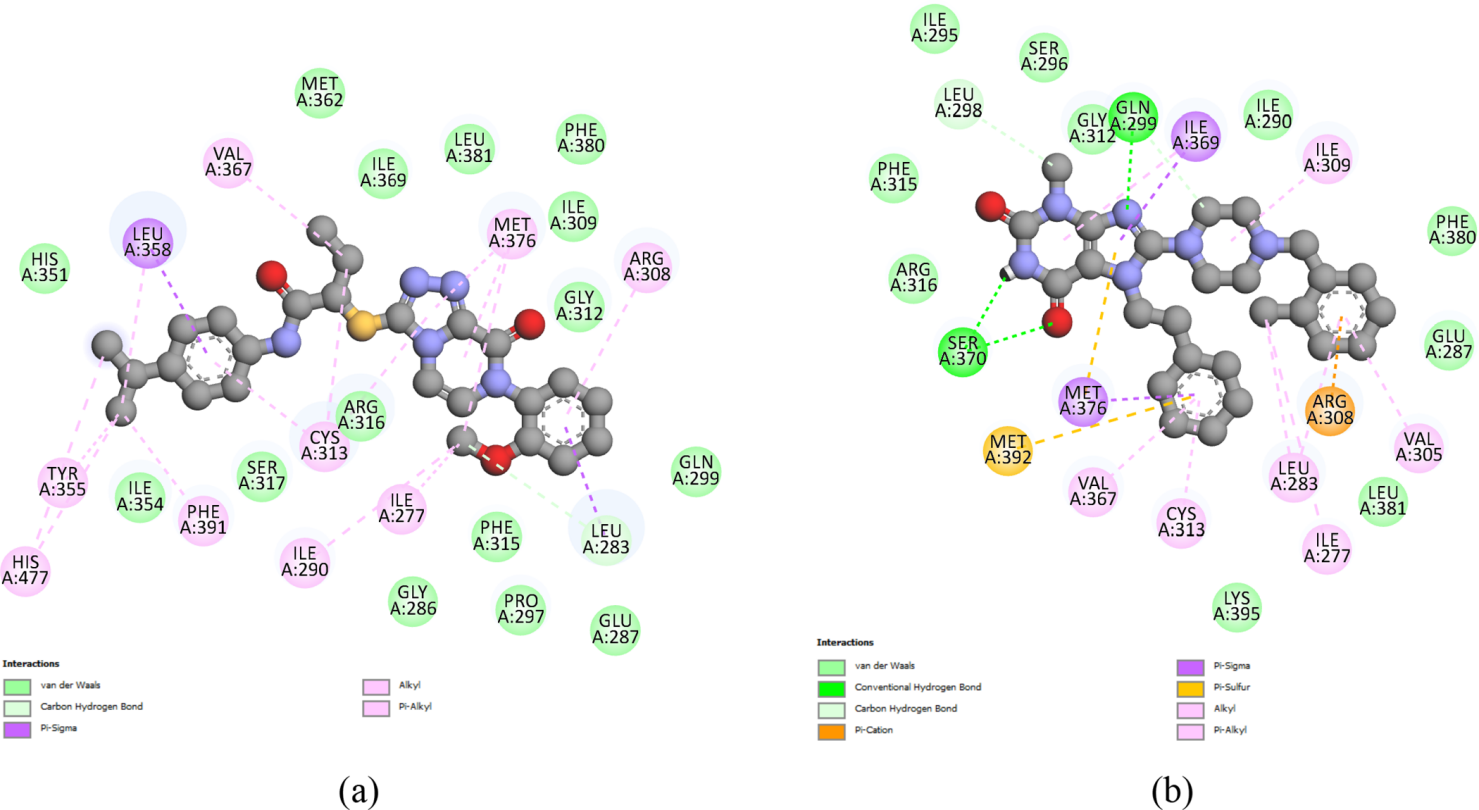
Interactions performed during 150 ns in molecular dynamics (MD) simulations for (a) Compound **3**/PPARγ and (b) Compound **4**/PPARγ. The diagrams were performed on frames of 21.35 and 43.64 ns, respectively.

In the case of the Compound **3**/PPARγ complex (Figure 14a), some interactions remained the same as the complex from the molecular *docking* calculation, such as the alkyl interactions of the hydrophobic ethyl branch with residues VAL-367 and CYS-313, and the methyl group of the ether function interacting with residue LEU-283 also through an alkyl interaction. The isopropyl of Compound **3** started to make new Pi–alkyl interactions with residues HIS-477, TYR-355, and PHE-391, something not predicted in the *docking* calculation, as shown in Figure 9a. The Pi–alkyl interaction of one of the aromatic rings with residue LEU-358 was converted to a Pi–sigma interaction. Interactions such as Pi–sulfur with the MET-392 residue, Pi–anion with the GLU-287 residue, and H-bond with the CYS-313 residue were not maintained after the molecular dynamics calculation.

In the case of the Compound **4**/PPARγ complex (Figure 14b), the residues responsible for the H-bonds in the molecular *docking* calculation were ARG-316 and GLU-319, while in molecular dynamics, they were GLN-299 and SER-370. The simulation results suggest that other intermolecular interactions, such as alkyl and Pi–alkyl interactions, and hydrophobic interactions involving several residues, including ILE-295, SER-296, PHE-380, LEU-381, and LYS-395, contribute significantly to the stabilization of Compound **4** in the PPARγ active site.

Compounds 3 and 4 occupy a binding site in PPARγ similar to that of rosiglitazone, as evidenced by several interactions with the same key residues. In addition, the molecular conformations adopt an almost horseshoe-like fold around helix H3. In particular, Compound 4 exhibits its piperazine region oriented toward helix H2, contributing to a slight displacement from H3.

Furthermore, molecular dynamics analysis revealed that both screened compounds establish hydrophobic and van der Waals interactions with key residues such as ILE-309, CYS-313, and MET-376. These are the same residues involved in PPARγ activation by rivoglitazone, which adopts a canonical full agonist binding mode, as reported by Harinda Rajapaksha et al.^55^ Consistent with the behavior of partial agonists described in the literature, Compounds **3** and **4** also interact hydrophobically with CYS-313 from helix H3 and form electrostatic or van der Waals interactions with ARG-316, as observed in the 2D interaction diagrams from the MD simulations. Furthermore, most partial agonists stabilize the β-sheet through hydrogen bonds between an acidic group and the backbone amine of SER-370. This same type of interaction was identified between the xanthine region of Compound **4** and residue SER-370, reinforcing its potential partial agonist character.^56^ Hydrogen bonding with SER-370 has also been reported for indole-based partial agonists such as SPPARγM2, MRL24, and nTZDpa,^57,58^ supporting the role of this residue in stabilizing partial binding modes.

Despite the presence of two hydrogen bonds between Compound **4** and PPARγ, both compounds, Compounds **3** and **4**, share partial similarities with the binding modes of GW0072 and GQ-16, which have been reported as ligands predominantly mediated by van der Waals and hydrophobic interactions. These interactions mainly involve residues ILE-309, CYS-313, ARG-316, ILE-354, LEU-358, and LEU-381.^59,60^

In the context of bovine lipid metabolism, such partial activation could fine-tune PPARγ-dependent adipogenic pathways in muscle cells, promoting moderate lipid deposition without excessive fat accumulation. This controlled adipogenesis aligns with the desirable marbling phenotype in beef production.

## 4 Conclusions

In conclusion, Compound **3** and Compound **4**, as alternative PPARγ activators, showed a predicted activity like that of the reference compound, rosiglitazone, as well as a higher theoretical LD_50_ value, raising expectations of new PPARγ activators that are safer than those currently on the market. This enhances the competitiveness of the agricultural market while also expanding the possibilities for using active ingredients as effective as the currently marketed active ingredient (rosiglitazone).

Although this study relies exclusively on computational approaches, experimental validation is crucial to confirm the predicted PPARγ activation potential of the identified compounds. Future investigations may employ biochemical and cellular assays to validate the in silico results. For instance, ligand-binding assays such as the in vitro competitive TR-FRET assay,^61^ fluorescence polarization assays,^62^ or reporter gene assays^63^ could be used to quantify ligand affinity and PPARγ-mediated transcriptional activity. Furthermore, adipocyte differentiation assays in preadipocyte cell lines such as 3T3-L1 could be conducted to evaluate receptor activation by monitoring lipid accumulation and the expression of adipogenesis-related target genes.^64^ Such experimental efforts would not only substantiate the computational findings but also strengthen the translational potential of these molecules as modulators of lipid metabolism and beef marbling.

## Abreviations

FAO: Food and Agriculture Organization
PPAR: peroxisome proliferator-activated receptor
IBMX: 3-isobutyl-1-methylxanthine
PPARγ: peroxisome proliferator-activated receptor gamma
ROC: receiver operating characteristic
NVT ensemble: moles (N), volume (V), and temperature (T)
NPT ensemble: moles (N), pressure (P), and temperature (T)
RMSD: root-mean-square deviation
RMSF: root-mean-square fluctuation
TVBL: virtual ligand-based screening
LD50: median lethal dose
AUC: area under the curve
RGZ: rosiglitazone
WLogP: log P method developed by Wildman and Crippen
TPSA: topological polar surface area
BBB: blood–brain barrier
HIA: human intestinal absorption
P-gp: P-glycoprotein

## References

[1] FAOSTAT. https://www.fao.org/faostat/en/#data (accessed Aug 30, 2023).

[2] Lage J. F., Paulino P. V. R., Filho S. C. V., Souza E. J. O., Duarte M. S., Benedeti P. D. B., Souza N. K. P., Cox R. B. (2012). Influence of Genetic
Type and Level of Concentrate in the Finishing Diet on Carcass and
Meat Quality Traits in Beef Heifers. Meat Sci.

[3] Rodrigues R. T. de S., Chizzotti M. L., Vital C. E., Baracat-Pereira M. C., Barros E., Busato K. C., Gomes R. A., Ladeira M. M., Martins T. da S. (2017). Differences in Beef Quality between Angus (Bos Taurus
Taurus) and Nellore (Bos Taurus Indicus) Cattle through a Proteomic
and Phosphoproteomic Approach. PLoS One.

[4] Viacava, C. Nelore: O Boi Ecológico Que Está Conquistando o Mundo; Peirópolis, 2000.

[5] Font-i-Furnols M., Guerrero L. (2014). Consumer Preference,
Behavior and Perception about
Meat and Meat Products: An Overview. Meat Sci.

[6] Polkinghorne R. J., Thompson J. M. (2010). Meat Standards and Grading. Meat
Sci.

[7] Costa E. C., Restle J., Brondani I. L., Perottoni J., Faturi C., Menezes L. F. (2002). Composição Física
Da Carcaça, Qualidade Da Carne e Conteúdo de Colesterol
No Músculo Longissimus Dorsi de Novilhos Red Angus Superprecoces,
Terminados Em Confinamento e Abatidos Com Diferentes Pesos. Rev. Bras. Zootec..

[8] Liu R., Liu X., Bai X., Xiao C., Dong Y. (2021). A Study of the Regulatory
Mechanism of the CB1/PPARγ2/PLIN1/HSL Pathway for Fat Metabolism
in Cattle. Front Genet.

[9] Hausman G. J., Dodson M. V., Ajuwon K., Azain M., Barnes K. M., Guan L. L., Jiang Z., Poulos S. P., Sainz R. D., Smith S., Spurlock M., Novakofski J., Fernyhough M. E., Bergen W. G. (2009). Board-Invited Review: The Biology
and Regulation of Preadipocytes and Adipocytes in Meat Animals. Journal of Animal Science.

[10] Rosen E. D., MacDougald O. A. (2006). Adipocyte
Differentiation from the inside Out. Nat Rev
Mol Cell Biol.

[11] Rosen E. D., Hsu C.-H., Wang X., Sakai S., Freeman M. W., Gonzalez F. J., Spiegelman B. M. (2002). C/EBPα Induces Adipogenesis
through PPARγ: A Unified Pathway. Genes
Dev..

[12] Calkin A. C., Jandeleit-Dahm K. A., Sebekova E., Allen T. J., Mizrahi J., Cooper M. E., Tikellis C. (2007). PPARs and Diabetes-Associated Atherosclerosis. Curr. Pharm. Des..

[13] Frohnert B. I., Hui T. Y., Bernlohr D. A. (1999). Identification of a Functional Peroxisome
Proliferator-Responsive Element in the Murine Fatty Acid Transport
Protein Gene. J. Biol. Chem..

[14] Lefterova M. I., Haakonsson A. K., Lazar M. A., Mandrup S. (2014). PPARγ and the
Global Map of Adipogenesis and Beyond. Trends
in Endocrinology & Metabolism.

[15] Kim H.-Y., Jang H.-J., Muthamil S., Shin U. C., Lyu J.-H., Kim S.-W., Go Y., Park S.-H., Lee H. G., Park J. H. (2024). Novel Insights into
Regulators and Functional Modulators
of Adipogenesis. Biomedicine & Pharmacotherapy.

[16] Tajnšek Š., Petrovič D., Petrovič M. G., Kunej T. (2020). Association of Peroxisome Proliferator-Activated
Receptors (PPARs)
with Diabetic Retinopathy in Human and Animal Models: Analysis of
the Literature and Genome Browsers. PPAR Res..

[17] Scott M. A., Nguyen V. T., Levi B., James A. W. (2011). Current
Methods
of Adipogenic Differentiation of Mesenchymal Stem Cells. Stem Cells Dev.

[18] Mitić R., Cantoni F., Börlin C. S., Post M. J., Jackisch L. (2023). A Simplified
and Defined Serum-Free Medium for Cultivating Fat across Species. iScience.

[19] Nissen S. E., Wolski K. (2007). Effect of Rosiglitazone on the Risk
of Myocardial Infarction
and Death from Cardiovascular Causes. New England
Journal of Medicine.

[20] Gampe R. T., Montana V. G., Lambert M. H., Miller A. B., Bledsoe R. K., Milburn M. V., Kliewer S. A., Willson T. M., Xu H. E. (2000). Asymmetry
in the PPARγ/RXRα Crystal Structure Reveals the Molecular
Basis of Heterodimerization among Nuclear Receptors. Mol. Cell.

[21] Zhang J., Wang Y., Wang Q., Vinayaka C. R., Ross K., Natale D. A., McGarvey P., Laiho K., Huang H., Chen Y., Chen C., Arminski L., Arighi C. N., Wu C. H., Sundaram S., Sonesson K., Sigrist C. J. A., Rivoire C., Redaschi N., Pruess M., Pozzato M., Poux S., Pourcel L., Pilbout S., Pedruzzi I., Paesano S., Muthukrishnan V., Morgat A., Masson P., Lieberherr D., Le Mercier P., Kerhornou A., Jungo F., Hyka-Nouspikel N., Hulo C., Gruaz N., Gos A., Gerritsen V., Gehant S., Gaudet P., Gasteiger E., Feuermann M., Famiglietti M. L., Estreicher A., de Castro E., Cuche B., Coudert E., Echioukh K. C., Casals-Casas C., Gil B. C., Breuza L., Boutet E., Bolleman J. T., Blatter M. C., Batista Neto T. M., Baratin D., Bansal P., Axelsen K. B., Auchincloss A. H., Argoud-Puy G., Aimo L., Bridge A. J., Zellner H., Zaru R., Watkins X., Warner K., Vasudev P., Tyagi N., Turner E., Totoo P., Stephenson J., Speretta E., Santos R., Saidi R., Rice D. L., Raposo P., Raj S., Qi G., Pundir S., Nightingale A., Moulang K., Mishra A., Mahmoudy M., Madeira F., MacDougall A., Lussi Y., Luo J., Lugaric M., Luciani A., Lock A., Kandasaamy S., Jyothi D., Joshi V., Ishtiaq R., Insana G., Ignatchenko A., Hussein A., Hatton-Ellis E., da Costa Gonzales L. J., Garmiri P., Fan J., Ebenezer T. G., Dogan T., Denny P., Cukura A., Bye-A-Jee H., Britto R., Bowler-Barnett E. H., Alpi E., Ahmad S., Magrane M., Orchard S., Martin M. J., Bateman A. (2023). UniProt: The
Universal Protein Knowledgebase in 2023. Nucleic
Acids Res..

[22] Waterhouse A., Bertoni M., Bienert S., Studer G., Tauriello G., Gumienny R., Heer F. T., De Beer T. A. P., Rempfer C., Bordoli L., Lepore R., Schwede T. (2018). SWISS-MODEL: Homology
Modelling of Protein Structures and Complexes. Nucleic Acids Res..

[23] Bienert S., Waterhouse A., De Beer T. A. P., Tauriello G., Studer G., Bordoli L., Schwede T. (2017). The SWISS-MODEL Repository-New
Features and Functionality. Nucleic Acids Res..

[24] Chen V. B., Arendall W. B., Headd J. J., Keedy D. A., Immormino R. M., Kapral G. J., Murray L. W., Richardson J. S., Richardson D. C. (2010). MolProbity: All-Atom Structure Validation
for Macromolecular
Crystallography. Acta Crystallogr., Sect. D:
Biol. Crystallogr..

[25] Studer G., Rempfer C., Waterhouse A. M., Gumienny R., Haas J., Schwede T. (2020). QMEANDisCo—Distance
Constraints Applied on Model
Quality Estimation. Bioinformatics.

[26] Lipinski C. A., Lombardo F., Dominy B. W., Feeney P. J. (2001). Experimental and
Computational Approaches to Estimate Solubility and Permeability in
Drug Discovery and Development Settings 1PII of Original Article:
S0169–409X(96)­00423–1. The Article Was Originally Published
in Advanced Drug Delivery Reviews 23 (1997) 3–25. 1. Adv Drug Deliv Rev.

[27] Daina A., Michielin O., Zoete V. (2017). SwissADME: A Free Web Tool to Evaluate
Pharmacokinetics, Drug-Likeness and Medicinal Chemistry Friendliness
of Small Molecules. Sci Rep.

[28] Lipinski C. A., Lombardo F., Dominy B. W., Feeney P. J. (2012). Experimental and
Computational Approaches to Estimate Solubility and Permeability in
Drug Discovery and Development Settings. Adv.
Drug Delivery Rev..

[29] Baell J. B., Holloway G. A. (2010). New Substructure
Filters for Removal of Pan Assay Interference
Compounds (PAINS) from Screening Libraries and for Their Exclusion
in Bioassays. J. Med. Chem..

[30] Brenk R., Schipani A., James D., Krasowski A., Gilbert I. H., Frearson J., Wyatt P. G. (2008). Lessons
Learnt from
Assembling Screening Libraries for Drug Discovery for Neglected Diseases. ChemMedChem.

[31] Trott O., Olson A. J. (2010). AutoDock Vina: Improving the Speed and Accuracy of
Docking with a New Scoring Function, Efficient Optimization, and Multithreading. J. Comput. Chem..

[32] Eberhardt J., Santos-Martins D., Tillack A. F., Forli S. (2021). AutoDock Vina 1.2.0:
New Docking Methods, Expanded Force Field, and Python Bindings. J. Chem. Inf. Model..

[33] Willson T. M., Cobb J. E., Cowan D. J., Wiethe R. W., Correa I. D., Prakash S. R., Beck K. D., Moore L. B., Kliewer S. A., Lehmann J. M. (1996). The Structure - Activity Relationship
between Peroxisome
Proliferator-Activated Receptor γ Agonism and the Antihyperglycemic
Activity of Thiazolidinediones. J. Med. Chem..

[34] Mysinger M. M., Carchia M., Irwin J. J., Shoichet B. K. (2012). Directory of Useful
Decoys, Enhanced (DUD-E): Better Ligands and Decoys for Better Benchmarking. J. Med. Chem..

[35] Pires D. E. V., Blundell T. L., Ascher D. B. (2015). PkCSM:
Predicting Small-Molecule
Pharmacokinetic and Toxicity Properties Using Graph-Based Signatures. J. Med. Chem..

[36] Hollingsworth S. A., Dror R. O. (2018). Molecular Dynamics Simulation for
All. Neuron.

[37] Karplus M., McCammon J. A. (2002). Molecular Dynamics Simulations of
Biomolecules. Nat. Struct. Biol..

[38] Schmid N., Eichenberger A. P., Choutko A., Riniker S., Winger M., Mark A. E., Van Gunsteren W. F. (2011). Definition and Testing of the GROMOS
Force-Field Versions 54A7 and 54B7. Eur. Biophys.
J..

[39] Darden T., Perera L., Li L., Pedersen L. (1999). New Tricks for Modelers
from the Crystallography Toolkit: The Particle Mesh Ewald Algorithm
and Its Use in Nucleic Acid Simulations. Structure.

[40] Bussi G., Donadio D., Parrinello M. (2007). Canonical Sampling through Velocity
Rescaling. J. Chem. Phys..

[41] Berendsen H. J. C., Postma J. P. M., van
Gunsteren W. F., DiNola A., Haak J. R. (1984). Molecular
Dynamics with Coupling to an External Bath. J. Chem. Phys..

[42] Parrinello M., Rahman A. (1980). Crystal Structure and
Pair Potentials: A Molecular-Dynamics
Study. Phys. Rev. Lett..

[43] Parrinello M., Rahman A. (1981). Polymorphic Transitions
in Single Crystals: A New Molecular
Dynamics Method. J. Appl. Phys..

[44] Yuan Y., Pei J., Lai L. (2013). Binding Site
Detection and Druggability Prediction
of Protein Targets for Structure- Based Drug Design. Curr. Pharm. Des..

[45] Wang S., Xie J., Pei J., Lai L. (2023). CavityPlus
2022 Update: An Integrated
Platform for Comprehensive Protein Cavity Detection and Property Analyses
with User-Friendly Tools and Cavity Databases. J. Mol. Biol..

[46] Geppert H., Vogt M., Bajorath J. (2010). Current Trends in Ligand-Based
Virtual
Screening: Molecular Representations, Data Mining Methods, New Application
Areas, and Performance Evaluation. J. Chem.
Inf. Model..

[47] Justin A., Mandal S., Prabitha P., Dhivya S., Yuvaraj S., Kabadi P., Sekhar S. J., Sandhya C. H., Wadhwani A. D., Divakar S., Bharathi J. J., Durai P., Prashantha
Kumar B. R. (2020). Rational Design, Synthesis, and In Vitro Neuroprotective
Evaluation of Novel Glitazones for PGC-1α Activation via PPAR-γ:
A New Therapeutic Strategy for Neurodegenerative Disorders. Neurotox. Res..

[48] Nanjan M. J., Mohammed M., Prashantha Kumar B. R., Chandrasekar M. J. N. (2018). Thiazolidinediones
as Antidiabetic Agents: A Critical Review. Bioorg.
Chem..

[49] Maia E. H. B., Assis L. C., de Oliveira T. A., da Silva A. M., Taranto A. G. (2020). Structure-Based
Virtual Screening: From Classical to Artificial Intelligence. Front Chem.

[50] Bell E. W., Zhang Y. (2019). DockRMSD: An Open-Source
Tool for Atom Mapping and RMSD Calculation
of Symmetric Molecules through Graph Isomorphism. J. Cheminform..

[51] Xue Y., Yap C. W., Sun L. Z., Cao Z. W., Wang J. F., Chen Y. Z. (2004). Prediction of P-Glycoprotein Substrates by a Support
Vector Machine Approach. J. Chem. Inf. Comput.
Sci..

[52] Schmitt L. (2002). Structure
and Mechanism of ABC Transporters. Curr Opin
Struct Biol.

[53] van Veen, H. W. ; Konings, W. N. Structure and Function of Multidrug Transporters. In Resolving the Antibiotic Paradox; Springer, 1998; pp 145–158. 10.1007/978-1-4615-4897-3_8 10549367

[54] Kim R. B., Fromm M. F., Wandel C., Leake B., Wood A. J., Roden D. M., Wilkinson G. R. (1998). The Drug
Transporter P-Glycoprotein
Limits Oral Absorption and Brain Entry of HIV-1 Protease Inhibitors. Journal of Clinical Investigation.

[55] Rajapaksha H., Bhatia H., Wegener K., Petrovsky N., Bruning J. B. (2017). X-Ray Crystal Structure of Rivoglitazone
Bound to PPARγ
and PPAR Subtype Selectivity of TZDs. Biochimica
et Biophysica Acta (BBA) - General Subjects.

[56] Kroker A. J., Bruning J. B. (2015). Review of the Structural
and Dynamic Mechanisms of
PPARγ Partial Agonism. PPAR Res.

[57] Einstein M., Akiyama T. E., Castriota G. A., Wang C. F., McKeever B., Mosley R. T., Becker J. W., Moller D. E., Meinke P. T., Wood H. B., Berger J. P. (2008). The Differential
Interactions of
Peroxisome Proliferator-Activated Receptor γ Ligands with Tyr473
Is a Physical Basis for Their Unique Biological Activities. Mol. Pharmacol..

[58] Bruning J. B., Chalmers M. J., Prasad S., Busby S. A., Kamenecka T. M., He Y., Nettles K. W., Griffin P. R. (2007). Partial Agonists Activate PPARγ
Using a Helix 12 Independent Mechanism. Structure.

[59] Oberfield J. L., Collins J. L., Holmes C. P., Goreham D. M., Cooper J. P., Cobb J. E., Lenhard J. M., Hull-Ryde E. A., Mohr C. P., Blanchard S. G., Parks D. J., Moore L. B., Lehmann J. M., Plunket K., Miller A. B., Milburn M. V., Kliewer S. A., Willson T. M. (1999). A Peroxisome Proliferator-Activated
Receptor γ Ligand Inhibits Adipocyte Differentiation. Proceedings of the National Academy of Sciences.

[60] Amato A. A., Rajagopalan S., Lin J. Z., Carvalho B. M., Figueira A. C. M., Lu J., Ayers S. D., Mottin M., Silveira R. L., Souza P. C. T., Mourão R. H. V., Saad M. J. A., Togashi M., Simeoni L. A., Abdalla D. S. P., Skaf M. S., Polikparpov I., Lima M. C. A., Galdino S. L., Brennan R. G., Baxter J. D., Pitta I. R., Webb P., Phillips K. J., Neves F. A. R. (2012). GQ-16,
a Novel Peroxisome Proliferator-Activated Receptor γ (PPARγ)
Ligand, Promotes Insulin Sensitization without Weight Gain. J. Biol. Chem..

[61] Lian Y.-E., Wang M., Ma L., Yi W., Liao S., Gao H., Zhou Z. (2024). Identification of Novel
PPARγ Partial Agonists
Based on Virtual Screening Strategy: In Silico and In Vitro Experimental
Validation. Molecules.

[62] Wang Y., Yao Y., Liu J., Wu L., Liu T., Cui J., Lee D. Y.-W. (2020). Synthesis and
Biological Activity of Piperine Derivatives
as Potential PPARγ Agonists. Drug Des.
Devel. Ther..

[63] Umeno A., Sakashita M., Sugino S., Murotomi K., Okuzawa T., Morita N., Tomii K., Tsuchiya Y., Yamasaki K., Horie M., Takahara K., Yoshida Y. (2020). Comprehensive Analysis
of PPARγ Agonist Activities of Stereo-, Regio-, and Enantio-Isomers
of Hydroxyoctadecadienoic Acids. Biosci. Rep..

[64] Flori L., Galgani G., Bray G., Ippolito C., Segnani C., Pellegrini C., Citi V., Bernardini N., Martelli A., Calderone V. (2025). Development of an Adipocyte Differentiation
Protocol Using 3T3-L1 Cells for the Investigation of the Browning
Process: Identification of the PPAR-γ Agonist Rosiglitazone
as a Browning Reference Drug. Front. Pharmacol..

